# Editorial: Plant Derived Products to Combat Bacterial, Fungal and Parasitic Pathogens

**DOI:** 10.3389/fvets.2020.570613

**Published:** 2020-10-27

**Authors:** Valentina Virginia Ebani, Abd El Nasser Gaber El Gendy, Francesca Mancianti

**Affiliations:** ^1^Department of Veterinary Sciences, University of Pisa, Pisa, Italy; ^2^Interdepartmental Research Center “Nutraceuticals and Food for Health”, University of Pisa, Pisa, Italy; ^3^Medicinal and Aromatic Plants Research Department, National Research Center, Giza, Egypt

**Keywords:** bacteria, fungi, parasites, plant derived products, antimicrobial activity

The livestock sector has a significant role in the global economies, mostly in the developing countries, providing energy, food, raw materials, and manure for crops. Wherefore, animal pathogens can determine serious social, economic, and environmental damage, as well as they can be cause of human diseases. In particular, several animal and human pathologies are caused by bacteria, fungi, and parasites.

In recent years, several conventional drugs have lost much of their effectiveness with consequent relevant side effects. The extensive use of antibiotics in veterinary medicine to treat bacterial infections, as well as for auxinic purpose, has strongly determined the spreading of antibiotic-resistant bacterial strains.

Fungal pathogens, mainly opportunistic, environmental species, show a decreased sensitivity to antimycotic drugs, probably due to the large use of fungicides in farming. Furthermore, the use of antibiotics and antimycotic conventional drugs may be cause of environmental contamination and presence of residues in food of animal origin (meat and poultry, milk and dairy products, eggs, fish and seafood, honey).

Moreover, endo and ectoparasites may have different degrees of resistance to conventional drugs, treatments may be frequently toxic and not always allowed in the treatment of production animals.

All these concerns may impact on human health, also. In view of this situation, the use of natural alternatives for livestock managing is welcome. Plant derived products have been suggested to have activity against bacterial, fungal, and parasitic pathogens. For this reason, they could be employed not only for therapeutic treatments, but also environmental hygiene and food preservation.

The present Research Topic shows the results of some studies which demonstrate the effectiveness of different natural products against pathogens responsible for diseases and lesions in farm and pet animals.

Propolis is a complex, resinous, and balsamic product produced by bees during the collection of resins from shoots, exudates, and other plant tissues. It contains additives such as salivary secretions, wax, and pollen (1). Several compounds have been identified in propolis: aliphatic acids and esters, aldehydes and aromatic esters, sugars, alcohols, fatty acids, amino acids, steroids, ketones, chalcones, flavonoids, terpenes, lignans, polyphenols, proteins, vitamins, and minerals (2). Several biological and pharmacological properties have been attributed to propolis, related to its chemical composition: antibacterial (3), antifungal (4), antiviral (5), antiparasitic (6), anti-inflammatory (7), healing (8), analgesic (9), immunomodulatory (10), hepatoprotective (11), antiulcerogenic (12), anticarcinogenic (6), and antioxidant (6).

Kalil et al. found the *in vitro* anti-biofilm and bactericide activity of a green propolis extract against *Corynebacterium pseudotuberculosis*. This agent is a bacterium frequently causing caseous lymphadenitis in sheep, a pathology for which surgical treatment is necessary. The authors have suggested to employ this extract in the post-surgical treatment of caseous lymphadenitis due to its positive effects on surgical wound healing, hair recovery, inhibition of wound contamination and bacterial growth (Kalil et al.).

Propolis has been demonstrated to have activity against fungi, too. In particular, Brazilian greeen, red, and brown propolis resulted *in vitro* effective against clinical isolates of *Malassezia pachydermatis*, a yeast frequently involved in cases of canine otitis and dermatitis (Deegan et al.).

Different plant derived products are supposed to have therapeutic activity. Essential oils obtained from plants are frequently employed for food flavoring and preservation as well as in cosmetic and pharmaceutical industries. Furthermore, they are largely used in folk medicine. Their antimicrobial properties against bacteria, virus, and fungi have been investigated and demonstrated for a long time (13).

The Amazonian plant *Libidibia ferrea* is commonly used in traditional medicine to treat inflammations, infections, and hyperglycemia. Formulations containing alcoholic extract of this plant resulted to have a beneficial activity on the wound healing in dogs. Américo et al. in fact, demonstrated the antimicrobial activity of this extract against *Staphylococcus aureus, Escherichia coli, Pseudomonas aeruginosa*, and *Candida krusei*. Moreover, a good dermal wound healing through wound fibroplasia was observed by the authors after treatment of dogs with this phytotherapic formulation.

Different natural products have showed antiparasitic activity, too. Tedesco et al. found Tomatine and 2′,4′-Dihydroxychalcone, two plant-derived compounds, as effective against the fish pathogens *Saprolegnia* spp. (Oomycota) and *Amyloodinium ocellatum* (Dinophyceae), which cause important losses in freshwater and marine aquaculture industry, respectively. The use of these natural compounds in the hatchery could be cost effective and safe for workers and environment, representing a good option to replace synthetic products in the control of these parasites. Even though *in vivo* investigations should be arranged to select doses of these compounds which can be effective against *A. ocellatum* and *Saprolegnia* spp, Tomatine and 2′,4′-Dihydroxychalcone could be a promising alternative active against the parasites but non-toxic for their marine or freshwater hosts, the environment and consumer.

Further plant derived products are available; for most of them studies have performed to determine their potential therapeutical properties. However, investigations to discover new compounds and verify their possible applications are necessary to find natural alternative for managing environmental hygiene, food preservation, and therapeutic treatments.

## Author Contributions

All authors listed have made a substantial, direct and intellectual contribution to the work, and approved it for publication.

## Conflict of Interest

The authors declare that the research was conducted in the absence of any commercial or financial relationships that could be construed as a potential conflict of interest.
